# Racial and Ethnic Disparities in Preoperative Surgical Wait Time and Renal Cell Carcinoma Tumor Characteristics

**DOI:** 10.3390/healthcare9091183

**Published:** 2021-09-08

**Authors:** Eduardo Quinonez-Zanabria, Celina I. Valencia, Waheed Asif, Jiping Zeng, Ava C. Wong, Alejandro Cruz, Juan Chipollini, Benjamin R. Lee, Francine C. Gachupin, Chiu-Hsieh Hsu, Ken Batai

**Affiliations:** 1 Department of Urology, University of Arizona, Tucson, AZ 85724, USA; equinone@email.arizona.edu (E.Q.-Z.); wasif1@email.arizona.edu (W.A.); jzeng1@arizona.edu (J.Z.); awong@email.arizona.edu (A.C.W.); acruz700@surgery.arizona.edu (A.C.); jchipollini@arizona.edu (J.C.); brlee@arizona.edu (B.R.L.); 2 University of Arizona Cancer Center, University of Arizona, Tucson, AZ 85724, USA; celina@arizona.edu; 3 Department of Family and Community Medicine, University of Arizona, Tucson, AZ 85724, USA; fcgachupin@arizona.edu; 4 Department of Epidemiology and Biostatistics, University of Arizona, Tucson, AZ 85724, USA; pchhsu@arizona.edu

**Keywords:** cancer health disparities, Latinos, American Indians, surgical disparities

## Abstract

Racial/ethnic minority groups have a disproportionate burden of kidney cancer. The objective of this study was to assess if race/ethnicity was associated with a longer surgical wait time (SWT) and upstaging in the pre-COVID-19 pandemic time with a special focus on Hispanic Americans (HAs) and American Indian/Alaska Natives (AIs/ANs). Medical records of renal cell carcinoma (RCC) patients who underwent nephrectomy between 2010 and 2020 were retrospectively reviewed (*n* = 489). Patients with a prior cancer diagnosis were excluded. SWT was defined as the date of diagnostic imaging examination to date of nephrectomy. Out of a total of 363 patients included, 34.2% were HAs and 8.3% were AIs/ANs. While 49.2% of HA patients experienced a longer SWT (≥90 days), 36.1% of Non-Hispanic White (NHW) patients experienced a longer SWT. Longer SWT had no statistically significant impact on tumor characteristics. Patients with public insurance coverage had increased odds of longer SWT (OR 2.89, 95% CI: 1.53–5.45). Public insurance coverage represented 66.1% HA and 70.0% AIs/ANs compared to 56.7% in NHWs. Compared to NHWs, HAs had higher odds for longer SWT in patients with early-stage RCC (OR, 2.38; 95% CI: 1.25–4.53). HAs (OR 2.24, 95% CI: 1.07–4.66) and AIs/ANs (OR 3.79, 95% CI: 1.32–10.88) had greater odds of upstaging compared to NHWs. While a delay in surgical care for early-stage RCC is safe in a general population, it may negatively impact high-risk populations, such as HAs who have a prolonged SWT or choose active surveillance.

## 1. Introduction

Kidney cancer is among the 10 most common cancers in the United States. Increased delays of elective surgeries due to the COVID-19 pandemic led to delays of urologic oncologic surgical treatments and necessitated the investigation of the impact of surgical delays on tumor pathologic characteristics and outcomes [1,2,3,4]. Studies suggest that patients with small kidney mass may be able to safely delay surgical treatment. However, little is known about drivers that influence surgical wait time (SWT) and the racial/ethnic differences in SWT on final tumor characteristics.

The purpose of this study was to assess if race/ethnicity was associated with longer SWT and upstaging at surgery in the pre-COVID-19 pandemic time. We focused on racial/ethnic disparities because renal cell carcinoma (RCC) disproportionately affects racial/ethnic minority groups, particularly Hispanic Americans (HAs) and American Indians and Alaska Natives (AIs/ANs) [5,6,7]. These racial/ethnic groups are also underrepresented in RCC health disparities research [8]. Disasters, such as hurricanes and the COVID-19 pandemic, have historically had a greater magnitude of negative effects on health status and healthcare access of racial/ethnic minority patients compared to non-Hispanic White (NHW) patients, and racial/ethnic minority patients may wait longer to receive cancer treatment [9,10,11]. COVID-19 has been found to be a driver for excess mortality among cancer patients, and it is unclear what this pattern looks like for racial/ethnic minorities in the U.S. [12].

## 2. Materials and Methods

Medical records of patients diagnosed with RCC who underwent nephrectomy between 2010 and 2020 (March 2020 before the COVID-19 pandemic worsened) at Banner-University Medical Center Tucson (Tucson, AZ, USA) were retrospectively reviewed (*n* = 489). Patients with a prior cancer diagnosis were excluded. Patients who had nephrectomy prior to 2010 and came back for additional surgical treatment were not included. The data collection methods have been previously described [13]. The study protocol was approved by the University of Arizona Institutional Review Board.

Due to geographic location (southern Arizona, USA), this study focused on HAs and AIs/ANs who also have a disproportionately higher burden of RCC [5]. Other racial/ethnic groups (African Americans and Asian Americans) that were underrepresented in the study (<5%), mixed-race individuals, and individuals without information on race/ethnicity were grouped together.

SWT was defined as the time period from the date of the first abnormal finding through imaging examinations (e.g., CT and MRI) to the date of nephrectomy. For an unknown date of imaging assessment, the date of the first encounter with a urologist or the date that a renal mass was found according to urologists’ clinic notes was used. If patients had bilateral RCC and had a nephrectomy on both sides of the kidney on different days, the date of the first nephrectomy was used. A longer SWT was defined as 90 days or more (≥90 days). Previous studies reported that more than 90 days of SWT was associated with poor overall survival in patients with a T2 renal tumor [14] and a renal tumor greater than 4 cm [15]. Increase in tumor size, the greatest dimension in centimeter (cm), was calculated using the tumor size measured during the initial imaging assessment and the size of the removed tumor. When the information on the initial imaging assessment was not available, the size estimated in the second imaging assessment was used. We also evaluated upstaging from clinical tumor stage 1 (cT1) based on tumor size at imaging assessment to pathological tumor stage 2 (pT2) or greater, lymph node positive (N1), or distant metastasis (M1) at the time of nephrectomy. For analysis of tumor size, we included only patients with detailed imaging records. When the imaging method was unknown, for example. when imaging assessment was done at other hospitals, the patients were excluded in the analysis related to tumor size.

Means and frequencies were calculated to summarize patient characteristics. A chi-squared test was conducted to assess the impacts of SWT on tumor characteristics and differences in clinicopathologic characteristics across racial/ethnic groups as well as between shorter and longer SWT. Logistic regression was performed to identify factors associated with longer SWT (≥90 days). Sensitivity analysis was performed by (1) removing patients with metastatic cancer, (2) stratifying based on tumor node metastasis (TNM) staging (early vs. advanced- stage), and (3) using a different cutoff for longer SWT (SWT < 75 vs. ≥75 days as well as <120 vs. ≥120 days). Logistic regression was also used to assess the association between race/ethnicity and upstaging adjusting for confounders including age, gender, binary SWT (<90 days vs. ≥90 days), marital status, insurance type, surgical year, body mass index (BMI), and smoking. IBM SPSS Statistics, version 27 (IBM Corp., Armonk, NY, USA) was used, and we consider two-side *p* < 0.05 as statistically significant.

## 3. Results

A total of 363 patients were deemed eligible for this study (Table 1). The median age of presentation was 59 years with males representing 62% of the total patient cohort. HAs and AIs/ANs were well represented (34.2% and 8.3%), while NHWs constituted 49.6% of the patients. The median SWT was 78 days (interquartile range from 45 to 134). The majority of patients were married (51.2%) and never smoked (56.2%). Hypertension was common (63.9%). Diabetes was more common in HAs (43.5%) and AIs/ANs (46.7%) than in NHWs (25.6%, *p* = 0.004; Supplementary Table S1). Public insurance coverage (Medicare, Medicaid, and Indian Health Services) was the most common type of insurance (60.6%). HAs (66.1%) and AIs/ANs (70.0%) were more likely to have public insurance coverage than NHWs (56.7%). The most common initial imaging assessment method was CT (63.6%) followed by MRI (9.6%). A partial nephrectomy (53.4%) and a robotic approach (46.1%) were more common surgical interventions. Clear cell RCC (83.2%) was the most frequent histological subtype, most commonly found in HAs (91.1%), followed by AIs/ANs (90%). The tumors were commonly Grade 3 or 4 (53.8%) and the American Joint Committee on Cancer TNM Stage I or II (63.5%).

Increase in tumor size (>2 cm) and up-staging were evaluated (Table 2). SWT did not have a significant impact on tumor size or upstaging. Tumor size increase was seen in 8.9% of patients with a shorter (<90 days) SWT and in 9.9% of patients with a longer (≥90 days) SWT. Pathological upstaging occurred in 25.7% of the patients with a shorter SWT and 26.4% of the patients with a longer SWT. When we focused on patients with a smaller tumor (≤7 cm), there was no difference.

Next, we assessed factors associated with a longer SWT (≥90 days). The representation of HAs was slightly higher in patients with a longer SWT (39.9%) compared to patients with a shorter SWT (30.0%, *p* = 0.14, Supplementary Table S2). Public insurance coverage comprised 53% of patients with a shorter SWT and 70.6% of patients with a longer SWT. Partial (62.1%) and robotic (54.2%) nephrectomies were common in patients with a longer SWT. On the other hand, high-grade and advanced-stage tumors were common in patients with a shorter SWT, and they were likely to undergo radical (42.4%) and open (46.9%) nephrectomies. Patients with public insurance were almost three times more likely to experience a longer SWT (OR, 2.89; 95% CI, 1.53–5.45) and were three times more likely after excluding patients with metastasis (OR 3.06; 95% CI, 1.59–5.87) (Table 3). The association between public insurance and longer SWT was significant even after stratifying for stage and using different cutoffs for longer SWT (Supplementary Tables S3 and S4). In patients with an advanced-stage tumor, public insurance had almost five-fold increased odds of a longer SWT (OR, 4.81; 95% CI 1.44–16.08). Among patients with an early-stage RCC, HAs had a two-fold increased odds of experiencing a longer SWT (OR, 2.38; 95% CI 1.25–4.53) but not in patients with an advanced-staged tumor. When the SWT ≥ 78 days was compared to <78 days (median SWT), HAs had statistically insignificant but increased odds of longer SWT (OR 1.62; 95% CI, 0.94–2.83).

Lastly, race/ethnicity was associated with upstaging. Upstaging was more common in HAs (27.8%) and AIs/ANs (38.5%) than NHWs (20.5%). HAs (OR 2.24, 95% CI: 1.07–4.66) and AIs/ANs (OR 3.79, 95% CI: 1.32–10.88) had increased odds of upstaging in the fully adjusted model (Table 4). Among patients with <90 days of SWT, HAs had significantly increased odds of upstaging (OR 4.19, 95% CI: 1.47–12.48), but not among patients with ≥90 days of SWT (OR 0.97, 95% C.I.: 0.26–3.64).

## 4. Discussion

This retrospective case series study did not find a negative effect of longer SWT on tumor characteristics at the time of nephrectomy in the overall study sample. However, this study focused on HAs and AIs/ANs who have high RCC incidence and mortality rates and found that HA patients in our cohort, who often have public insurance, experienced a longer SWT and had increased odds for upstaging. AIs/ANs also had increased odds of upstaging. Timely referral and treatment for these high-risk patients who face multiple barriers to healthcare may be recommended.

The relationship between a delay of surgical treatment of patients with RCC, particularly for smaller tumors, and oncological outcomes is well-documented in the literature and has been shown to be relatively safe [2,3]. RCC generally grows slowly, and active surveillance is a safe option for patients with a small renal mass without adverse oncologic outcomes [16,17]. Reviews by Tachibana et al. [4] and Katims et al. [1] have both demonstrated that delaying urological treatment is generally safe in patients with low-risk tumor characteristics. These findings were also confirmed by a recent review by Srivastava et al. [3] that found little risk for renal tumor upstaging. It is worth noting that extending treatment beyond five to six months in patients with T2 renal tumors was found to have poor overall survival compared to shorter SWT [14]. Another study reported that more than 90 days of SWT was associated with poor overall survival in patients with a renal tumor greater than 4 cm [15].

Although longer SWT is relatively safe, there are subgroups of patients likely to have longer SWT or choose active surveillance. Insurance coverage is one of the major factors for cancer treatment access that can prolong time to treatment initiation [18,19]. Our study findings on a longer SWT for patients with public insurance aligns with previous studies for other types of cancers, such as prostate and breast cancer, showing that treatment delays for patients are pronounced among racial/ethnic minority patients with public insurance [20,21,22]. The prior authorization requirement by public insurance and/or high deductible likely affects timely access to necessary care [23,24], but the driver of delays in treatment or choosing active surveillance for patients with public insurance is unclear. Further research is needed to better understand the RCC treatment delay experienced and selecting active surveillance by race/ethnic minorities who have public insurance.

When compared to NHWs, racial/ethnic minority patients face barriers in access to opportunities in nearly all facets of life. Structural racism plays out in education, employment, healthcare, and other key areas that impact disparate healthcare access, including health insurance coverage and availability of well-qualified primary care providers and specialty cares, and health outcomes among racial/ethnic minorities [25,26,27]. Even before the COVID-19 pandemic, the data demonstrate a delay of care for cancer patients occurring among racial/ethnic minority groups [28,29]. The drivers for delays in seeking healthcare despite experiencing symptoms range from medical distrust, low perceived symptoms that the individual expected to resolve over time, and barriers to healthcare access such as lack of insurance [28,30,31]. HAs are the highest-ranking population for being uninsured [32]. The lack of health insurance among HAs is an established factor in lower rates of cancer screenings, receiving a diagnosis at a later stage, and treatment [33]. Additionally, HAs often experience cancer treatment delays across various cancer types [34,35]. Medical mistrust and healthcare dissatisfaction have been found to serve as a salient factor for AI/AN cancer patients, and access to specialized cancer care, cancer information, and culturally competent care is limited for AI/AN patients [36]. When access to healthcare is available, the literature indicates a delay to surgery and other treatments as a salient barrier. The disparity of delayed treatment for AI/AN compared to the NHW populations has been found among insured AI/AN groups as well [28].

Moreover, subgroups of patients may also have a higher risk of upstaging at the time of nephrectomy. The literature has shown that African Americans have reduced odds of upstaging compared to NHWs, while patients with comorbidity have increased odds of upstaging [37,38]. This study reports that HAs and AIs/ANs who often have delayed care were at greatest risk for renal tumor upstaging. Co-morbidities, including obesity and diabetes, were very common among HA and AI/AN patients in our study. We have previously shown persistent oncological health disparities and mortality rates among AIs/ANs and HAs, especially U.S.-born Mexican Americans in Arizona [5,13]. While a longer SWT and active surveillance are safe for the general population with low risk, the longer SWT may not be safe for high-risk patients, such as HAs and AIs/ANs.

There were several limitations to this study, including the small sample size at a single institution limiting the generalizability of the study. Due to our geographic location, African Americans who experience marked RCC health disparities were not well-represented. Follow-up data including tumor recurrence and mortality were unavailable. This study evaluated the impact of a long interval from the time of abnormal finding on imaging to surgical treatment, but some patients may have experienced symptoms long before the imaging assessment or may have navigated a lengthy referral and insurance approval process to receive imaging assessment. In many cases, it was not clear if patients who had a long SWT chose active surveillance from medical records. Additionally, many other factors that may impact SWT, such as cardiac comorbidity, performance status, and cultural values, were not incorporated into our analysis. Information on recurrence and mortality was not collected as a part of this study, and we were not able to evaluate the long-term effects of prolonged SWT. However, this study is unique and has a high representation of racial/ethnic minority groups, such as HAs and AIs/ANs with high RCC mortality, that have been previously underrepresented in RCC studies [5,8,39]. In our state, a large part of the state is categorized as medically underserved, including areas with high proportions of racial/ethnic minority residents. This complicates access to urology care, radiology examination, surveillance, and surgical and oncology treatment for HA and AI/AN patients [5]. This may have impacted the composition of the study sample.

## 5. Conclusions

While a delay in surgical care for early-stage RCC and active surveillance of small renal tumors are safe in a general population, they may negatively impact high-risk populations, such as HAs who tend to have a longer SWT or may choose an active surveillance of small kidney mass. The disparities in the SWT among racial/ethnic minority groups warrant further investigations.

## Figures and Tables

**Table 1:** Characteristics of patients included in the study.

Variable	*n* (%) ^1^
Age at Presentation, Median (IQR)	59 (49–67)
Gender	
Male	225 (62.0)
Female	138 (38.0)
Race/Ethnicity	
Non-Hispanic Whites	180 (49.6)
Hispanic Americans	124 (34.2)
American Indians/Alaska Natives	30 (8.3)
Others/Mixed/Unknown	29 (8.0)
Marital Status	
Married	186 (51.2)
Not married	150 (41.3)
Unknown	27 (7.4)
Insurance Type	
Private	82 (22.6)
Public	220 (60.6)
No insurance	17 (4.7)
Unknown	44 (12.1)
Smoking	
Never smoked	203 (56.2)
Former smoker	96 (26.6)
Current smoker	62 (17.2)
Body Mass Index	
<25	54 (15.0)
≥25, <30	105 (29.1)
≥30, <35	102 (28.3)
≥35	100 (27.7)
Hypertension	
No	131 (36.1)
Yes	232 (63.9)
Diabetes	
No	241 (66.4)
Yes	122 (33.6)
Family History of Kidney Cancer	
No	335 (97.4)
Yes	9 (2.6)
Imaging Type	
CT	231 (63.6)
MRI	35 (9.6)
Ultrasound	58 (16.0)
X-ray	3 (0.8)
Unknown	36 (9.9)
Tumor Size (cm) at Imaging Assessment, Median (IQR)	4.6 (2.9–6.6)
Surgery Wait Time (days), Median (IQR)	78 (45–134)
Nephrectomy Type	
Partial	194 (53.4)
Radical	135 (37.2)
Cytoreductive	34 (9.4)
Surgical Approach	
Robotic	167 (46.1)
Laparoscopic	48 (13.3)
Open	147 (40.6)
Histologic Subtype	
Clear cell	302 (83.2)
Papillary	37 (10.2)
Chromophobe	12 (3.3)
Mixed/other/unspecified	12 (3.3)
Pathology Tumor Size (cm), Median (IQR)	4.5 (2.9–7.0)
Grade	
1 or 2	163 (46.2)
3 or 4	190 (53.8)
TNM Stage	
I or II	230 (63.5)
III or IV	132 (36.5)

^1^ *n* (%) for categorial variables and median and inter-quartile range (IQR) for continuous variables.

**Table 2:** Impact of surgical wait time on tumor characteristics.

Variable	<90 Days	≥90 Days	*p*
All, *n* (%)	210 (57.9)	153 (42.1)	
Tumor size increased ≥ 2 cm			0.85
No	174 (91.1)	127 (90.1)	
Yes	17 (8.9)	14 (9.9)	
Upstaged from cT1 to pT2 or greater, N1, or M1, *n* (%)			0.99
No	101 (74.3)	92 (73.6)	
Yes	35 (25.7)	33 (26.4)	
Patients with cT1 tumor (≤7 cm), *n* (%)	135 (50.7)	125 (48.1)	
Tumor size increased ≥2 cm			0.67
No	123 (91.8)	111 (89.5)	
Yes	11 (8.2)	13 (10.5)	
Upstaged from cT1 to pT2 or greater, N1, or M1			0.89
No	101 (74.8)	92 (72.6)	
Yes	34 (25.2)	33 (26.4)	

**Table 3:** Factors associated with more than 90 days SWT in a logistic regression analysis.

Variable	All		Without Metastasis	
	OR (95% C.I.)	*p*	OR (95% C.I.)	*p*
Age at Presentation		0.07		0.047
<50	Reference		Reference	
≥50, <65	1.47 (0.81–2.66)		1.44 (0.78–2.65)	
≥65	0.74 (0.39–1.41)		0.66 (0.24–1.28)	
Gender		0.70		0.46
Male	Reference		Reference	
Female	1.10 (0.68–1.78)		1.21 (0.73–2.01)	
Race/Ethnicity		0.51		0.68
Non-Hispanic Whites	Reference		Reference	
Hispanic Americans	1.43 (0.84–2.42)		1.34 (0.77–2.34)	
American Indians/Alaska Natives	1.06 (0.45–2.47)		0.86 (0.35–2.14)	
Others/Mixed/Unknown	1.59 (0.65–3.87)		1.05 (0.40–2.80)	
Insurance		0.01		0.009
Private	Reference		Reference	
Public	2.89 (1.53–5.45)		3.06 (1.59–5.87)	
No insurance	1.86 (0.53–6.56)		1.70 (0.43–6.64)	
Unknown	2.55 (0.98–6.64)		2.48 (0.14–0.56)	
Tumor Size at Imaging Assessment		<0.001		<0.001
≤7 cm	Reference		Reference	
>7 cm	0.27 (0.15–0.52)		0.28 (0.14–0.56)	
Surgery Year		0.02		0.01
2010–2012	Reference		Reference	
2013–2015	0.93 (0.46–1.88)		0.99 (0.48–2.06)	
2016–2020	2.05 (1.12–3.78)		2.22 (1.19–4.15)	

**Table 4:** Association between race/ethnicity and odds of upstaging.

Race/Ethnicity	Unadjusted		Adjusted Model 1		Adjusted Model 2	
	OR (95% C.I.)	*p*	OR (95% C.I.)	*p*	OR (95% C.I.)	*p*
Non-Hispanic Whites	Reference	0.13	Reference	0.053	Reference	0.02
Hispanic Americans	1.49 (0.80–2.81)		2.00 (0.99–4.04)		2.24 (1.07–4.66)	
American Indians/Alaska Natives	2.43 (0.99–65.97)		2.89 (1.07–7.81)		3.79 (1.32–10.88)	
Others/Mixed/Unknown	2.47 (0.87–7.00)		3.29 (1.05–10.26)		4.41 (1.32–14.73)	

Adjusted Model 1 includes race/ethnicity. Age, gender, SWT (<90 days vs. ≥90 days), surgical year, and BMI. Adjusted Model 2 additionally adjusts for marital status, insurance type, and smoking.

## Data Availability

The data used in the current study is available upon request.

## Supplementary Materials

The following are available online at https://www.mdpi.com/article/10.3390/healthcare9091183/s1, Table S1: Comparison of demographic and clinical characteristics across racial/ethnic groups, Table S2: Comparison of patients who had less than 90 days and more than 90 days SWT and unadjusted logistic regression analysis results for demographic and pre-surgical clinical information, Table S3: Factors associated with more than 90 days SWT stratified by TNM stage, Table S4: Sensitivity analysis for factors associated with longer SWT.
