# Supplementary material for: Racial and Ethnic Disparities in Preoperative Surgical Wait Time and Renal Cell Carcinoma Tumor Characteristics
Source: Healthcare (Basel). 2021 Sep 8;9(9):1183. doi: 10.3390/healthcare9091183 (PMC8471651; doi:10.3390/healthcare9091183)
Supplement: Supplementary file 1 [file healthcare-09-01183-s001.zip › healthcare-1333437-supplementary.pdf]

Supplementary Materials

Table S1. Comparison of demographic and clinical characteristics across racial/ethnic groups.

Variable	NHW	HA	AI/AN	Others/Unknown	*p*
n (%)	180 (49.6)	124 (34.2)	30 (8.3)	29 (8.0)	
Age at Presentation, Median (IQR)	62 (53-69)	55 (48-64)	55 (40-61)	59 (53-67)	<0.001
Sex, n (%)					0.59
Male	116 (64.4)	71 (57.3)	20 (66.7)	18 (62.1)	
Female	64 (35.6)	53 (42.7)	10 (33.3)	11 (37.9)	
Surgical Wait Time (≥90 days), n (%)					0.14
<90 days	115 (63.9)	63 (50.8)	16 (53.3)	16 (55.2)	
≥90 days	65 (36.1)	61 (49.2)	14 (46.7)	13 (44.8)	
Marital Status, n (%)					0.001
Married	98 (54.4)	65 (52.4)	9 (30.0)	14 (48.3)	
Widowed	11 (6.1)	10 (8.1)	1 (3.3)	2 (6.9)	
Single	41 (22.8)	28 (22.6)	19 (63.3)	6 (20.7)	
Divorced	15 (8.3)	15 (12.1)	0 (0.0)	2 (6.9)	
Unknown	15 (8.3)	6 (4.8)	1 (1.1)	5 (17.2)	
Smoking, n (%)					0.01
Never smoked	84 (46.9)	81 (65.3)	18 (62.1)	20 (69.0)	
Former smoker	56 (31.3)	31 (25.0)	5 (17.2)	4 (13.8)	
Current Smoker	39 (21.8)	12 (9.7)	6 (20.7)	5 (17.2)	
Insurance, n (%)					0.16
Private	46 (25.6)	28 (22.6)	3 (10.0)	5 (17.2)	
Public	102 (56.7)	82 (66.1)	21 (70.0)	15 (51.7)	
No coverage	8 (4.4)	6 (4.8)	1 (3.3)	2 (6.9)	
Unknown	24 (13.3)	8 (6.5)	5 (16.7)	7 (24.1)	
Body Mass Index, n (%)					0.06
<25	34 (18.9)	15 (12.2)	3 (10.0)	2 (7.1)	
≥25, <30	58 (32.2)	29 (23.6)	6 (20.0)	12 (42.9)	
≥30, <35	47 (26.1)	41 (33.3)	7 (23.3)	7 (25.0)	
≥35	41 (22.8)	38 (30.9)	14 (46.7)	7 (25.0)	
Hypertension, n (%)					0.50
No	64 (35.6)	45 (36.3)	14 (46.7)	6 (27.6)	
Yes	116 (64.4)	79 (63.7)	16 (53.3)	21 (72.4)	
Diabetes, n (%)					0.004
No	134 (74.4)	70 (56.5)	16 (53.3)	21 (72.4)	
Yes	46 (25.6)	54 (43.5)	14 (46.7)	8 (27.6)	
Family History of Kidney cancer, n (%)					0.75
No	168 (97.7)	115 (96.6)	25 (96.2)	27 (100.0)	
Yes	4 (2.3)	4 (3.4)	1 (3.8)	0 (0.0)	
Age at Surgery, Median (SD)	62 (53-69)	56 (48-64)	55 (40-61)	60 (53-67)	<0.001
Histological Subtype, n (%)					0.001
Clear cell	143 (80.3)	113 (91.1)	27 (90.0)	18 (62.1)	
Papillary	24 (13.5)	7 (5.6)	0 (0.0)	6 (3.4)	
Chromophobe	7 (3.9)	3 (2.4)	1 (3.3)	1 (3.4)	
Mixed subtype	2 (1.1)	1 (0.8)	2 (6.7)	2 (6.9)	
Others	2 (1.1)	0 (0.0)	0 (0.0)	2 (6.9)	
Grade, n (%)					0.55
1 or 2	77 (44.3)	61 (50.8)	14 (46.7)	11 (37.9)	
3 or 4	97 (55.7)	59 (49.2)	16 (53.3)	18 (62.1)	

TNM Stage, n (%)					0.09
I or II	123 (68.7)	77 (62.1)	16 (53.3)	14 (48.3)	
III or IV	56 (31.3)	47 (37.9)	14 (46.7)	15 (51.7)	
Upstaged from cT1 to pT2 or greater, N1, or M1, n (%)					0.12
No	101 (79.5)	65 (72.2)	16 (61.5)	11 (61.1)	
Yes	26 (20.5)	25 (27.8)	10 (38.5)	7 (38.9)	

Table S2. Comparison of patients who had less than 90 days and more than 90 days SWT and unadjusted logistic regression analysis results for demographic and pre-surgical clinical information.

**Variable**	**<90 days**	**≥ 90 days**	***p***	**OR (95% C.I.)**	***p***
Age at Presentation, n (%)			0.62		0.62
<50	52 (24.8)	40 (26.1)		Reference	
≥50, <65	86 (41.0)	68 (44.4)		1.03 (0.61-1.73)	
≥65	72 (34.3)	45 (29.4)		0.81 (0.47-1.42)	
Gender, n (%)			0.58		0.54
Male	133 (63.3)	92 (60.1)		Reference	
Female	77 (36.7)	61 (39.9)		0.87 (0.57-1.34)	
Race/Ethnicity, n (%)			0.14		0.14
Non-Hispanic Whites	115 (54.8)	65 (42.5)		Reference	
Hispanic Americans	63 (30.0)	61 (39.9)		1.71 (1.08-2.73)	
American Indians/Alaska Natives	16 (7.6)	14 (9.2)		1.55 (0.71-3.37)	
Others/Mixed/Unknown	16 (7.6)	13 (8.5)		1.44 (0.65-3.18)	
Marital Status, n (%)			0.20		0.20
Married	116 (55.2)	70 (45.5)		Reference	
Not married	79 (37.6)	71 (46.4)		1.49 (0.96-2.31)	
Unknown	15 (7.1)	12 (7.8)		1.33 (0.59-3.00)	
Insurance, n (%)			0.009		0.01
Private	58 (27.6)	24 (15.7)		Reference	
Public	112 (53.3)	108 (70.6)		2.33 (1.35-4.02)	
No insurance	12 (5.7)	5 (3.3)		1.01 (0.32-3.17)	
Unknown	28 (13.3)	16 (10.5)		1.38 (0.64-3.00)	
Smoking, n (%)			0.38		0.38
Never smoked	124 (59.3)	79 (52.0)		Reference	
Former smoker	52 (24.9)	44 (28.9)		1.33 (0.81-2.17)	
Current smoker	33 (15.8)	29 (19.1)		1.38 (0.78-2.45)	
Body Mass Index, n (%)			0.65		0.65
<25	29 (13.9)	25 (16.4)		Reference	
≥25, <30	66 (31.6)	39 (25.7)		0.69 (0.35-1.33)	
≥30, <35	58 (27.8)	44 (28.9)		0.88 (0.45-1.71)	
≥35	56 (26.8)	44 (28.9)		0.91 (0.47-1.77)	
Hypertension, n (%)			0.83		0.79
No	77 (36.7)	54 (35.3)		Reference	
Yes	133 (63.3)	99 (64.7)		1.06 (0.69-1.64)	
Diabetes, n (%)			0.31		0.30
No	144 (68.6)	97 (63.4)		Reference	
Yes	66 (31.4)	56 (36.6)		1.26 (0.81-1.96)	
Family History of Kidney Cancer, n (%)			0.74		0.63
No	196 (97.0)	139 (97.9)		Reference	
Yes	6 (3.0)	3 (2.1)		0.71 (0.17-2.87)	

Imaging Type, n (%)			0.43		0.33
CT	142 (67.6)	89 (58.2)		Reference	
MRI	18 (8.6)	17 (11.1)		1.51 (0.74-3.08)	
Ultrasound	30 (14.3)	28 (18.3)		1.54 (0.88-2.73)	
X-ray	1 (0.5)	2 (1.3)		1.43 (0.71-2.89)	
Unknown	19 (9.0)	17 (11.1)			
Tumor Size at Imaging Assessment (cm), n (%)			<0.001		<0.001
≤7 cm	135 (70.3)	126 (88.1)		Reference	
>7 cm	57 (29.7)	17 (11.9)		0.32 (0.18-0.58)	
Surgery Year			0.001		0.002
2010-2012	85 (40.5)	42 (27.5)		Reference	
2013-2015	51 (24.3)	28 (18.3)		1.11 (0.62-2.01)	
2016-2020	74 (35.2)	83 (54.2)		2.27 (1.40-3.69)	
Nephrectomy Type, n (%)			0.02		
Partial	99 (47.1)	95 (62.1)			
Radical	89 (42.4)	46 (30.1)			
Cytoreductive	22 (10.5)	12 (7.8)			
Surgical Approach, n (%)			0.01		
Robotic	84 (40.2)	83 (54.2)			
Laparoscopic	27 (12.9)	21 (13.7)			
Open	98 (46.9)	49 (32.0)			
Histologic Subtype, n (%)			0.45		
Clear cell	169 (80.5)	133 (86.9)			
Papillary	25 (11.9)	12 (7.8)			
Chromophobe	8 (3.8)	4 (2.6)			
Mixed/other/unspecified	8 (3.8)	4 (2.6)			
Pathology Tumor Size (cm), Median (IQR)	5.0 (3.5-8.1)	4.0 (2.7-5.5)	<0.001		
Grade, n (%)			0.02		
1 or 2	85 (41.1)	78 (53.4)			
3 or 4	122 (58.9)	68 (46.6)			
TNM Stage, n (%)			0.03		
I or II	123 (58.6)	107 (70.4)			
III or IV	87 (41.4)	45 (29.6)			

Table S3. Factors associated with more than 90 days SWT stratified by TNM stage.

**Variable**	**Early-Stage**		**Advanced-Stage**	
	**OR (95% C.I.)**	***p***	**OR (95% C.I.)**	***p***
Age at Presentation		0.49		0.54
<50	Reference		Reference	
≥50, <65	1.43 (0.71-2.88)		0.89 (0.32-2.47)	
≥65	1.02 (0.47-2.21)		0.74 (0.39-1.41)	
Gender, n (%)		0.54		0.30
Male	Reference		Reference	
Female	1.20 (0.68-2.13)		0.65 (0.29-1.47)	
Race/Ethnicity, n (%)		0.06		0.34
Non-Hispanic Whites	Reference		Reference	
Hispanic Americans	2.38 (1.25-4.53)		0.76 (0.31-1.91)	
American Indians/Alaska Natives	1.01 (0.33-3.08)		1.74 (0.47-6.45)	
Others/Mixed/Unknown	0.97 (0.31-3.06)		2.33 (0.63-8.66)	

Insurance, n (%)		0.11		0.01
Private	Reference		Reference	
Public	2.19 (1.06-4.54)		4.81 (1.44-16.08)	
No insurance	1.27 (0.26-6.13)		2.08 (0.26-16.48)	
Unknown	3.18 (1.21-9.62)		1.38 (0.27-7.12)	
Surgery Year		0.04		0.37
2010-2012	Reference		Reference	
2013-2015	1.47 (0.64-3.39)		0.71 (0.21-2.34)	
2016-2020	2.47 (1.21-5.08)		1.43 (0.47-4.38)	

Table S4. Sensitivity analysis for factors associated with longer SWT.

	**SWT <78 vs ≥ 78 days**		**SWT <120 vs ≥ 120 days**	
	**OR (95% C.I.)**	***p***	**OR (95% C.I.)**	***p***
Age at Presentation		0.03		0.17
<50	Reference		Reference	
≥50, <65	1.68 (0.89-3.15)		1.26 (0.68-2.33)	
≥65	0.72 (0.37-1.43)		0.69 (0.35-1.36)	
Gender, n (%)		0.89		0.70
Male	Reference		Reference	
Female	1.04 (0.62-1.74)		1.10 (0.67-1.83)	
Race/Ethnicity, n (%)		0.39		0.51
Non-Hispanic Whites	Reference		Reference	
Hispanic Americans	1.62 (0.94-2.83)		1.20 (0.68-2.12)	
American Indians/Alaska Natives	1.47 (0.58-3.72)		1.25 (0.52-3.00)	
Others/Mixed/Unknown	1.30 (0.51-3.35)		2.05 (0.83-5.06)	
Marital Status, n (%)		0.04		
Married	Reference			
Not married	1.84 (1.46-5.35)			
Unknown	2.51 (0.80-7.88)			
Insurance, n (%)		0.005		0.054
Private	Reference		Reference	
Public	2.80 (1.46-5.35)		2.56 (1.28-5.10)	
No insurance	0.66 (0.16-2.69)		3.01 (0.83-10.92)	
Unknown	1.47 (0.51-4.21)		2.46 (0.86-7.03)	
Imaging Type, n (%)		0.03		
CT	Reference			
MRI	1.86 (0.80-4.34)			
Ultrasound or X-ray	1.79 (0.93-3.45)			
Unknown	4.22 (1.33-13.41)			
Surgery Year		0.047		0.19
2010-2012	Reference		Reference	
2013-2015	0.79 (0.39-1.63)		1.26 (0.59-2.69)	
2016-2020	1.71 (0.91-3.22)		1.81 (0.93-3.52)	
Tumor Size at Imaging Assessment		<0.001		0.001
≤7 cm	Reference		Reference	
>7 cm	0.17 (0.09-0.33)		0.30 (0.15-0.62)	
